# A Review of SMA-Based Actuators for Bidirectional Rotational Motion: Application to Origami Robots

**DOI:** 10.3389/frobt.2021.678486

**Published:** 2021-07-02

**Authors:** Kejun Hu, Kanty Rabenorosoa, Morvan Ouisse

**Affiliations:** Université Bourgogne Franche-Comté, FEMTO-ST Institute, CNRS/UFC/ENSMM/UTBM, Besançon, France

**Keywords:** shape memory alloy, origami robot, shape-changing, modeling, bi-directional rotational motion

## Abstract

Shape memory alloys (SMAs) are a group of metallic alloys capable of sustaining large inelastic strains that can be recovered when subjected to a specific process between two distinct phases. Regarding their unique and outstanding properties, SMAs have drawn considerable attention in various domains and recently became appropriate candidates for origami robots, that require bi-directional rotational motion actuation with limited operational space. However, longitudinal motion-driven actuators are frequently investigated and commonly mentioned, whereas studies in SMA-based rotational motion actuation is still very limited in the literature. This work provides a review of different research efforts related to SMA-based actuators for bi-directional rotational motion (BRM), thus provides a survey and classification of current approaches and design tools that can be applied to origami robots in order to achieve shape-changing. For this purpose, analytical tools for description of actuator behaviour are presented, followed by characterisation and performance prediction. Afterward, the actuators’ design methods, sensing, and controlling strategies are discussed. Finally, open challenges are discussed.

## 1 Introduction

Shape memory alloys (SMAs)^1^ are a group of metallic alloys capable of sustaining large inelastic strains that can be recovered when subjected to a specific process between two distinct phases, which is temperature or magnetic field dependent. Two key behaviors of SMAs result from this transformation: shape memory effect (SME) and pseudoelasticity (PE) (Shaw et al., 2008). The former refers to the material’s ability to recover large, seemingly permanent strains via thermal stimulus from a deformed shape in martensite to a “memorized” one. The latter is associated with SMAs being able to undergo large, hysteretic stress-strain excursions without any permanent deformations at a sufficiently high temperature (Lester et al., 2015). Because of its 1) high energy density, 2) reasonable operational strain (Wei et al., 1998) relying on SME, 3) bio-compatibility, 4) and its long life^2^ (Ikuta, 1990), the SMAs provide a good potential of development of advanced and inexpensive actuators (see Table 1), which could significantly reduce the mechanical complexity and size of structures. Over the last years, the demand for SMAs for engineering and technical applications has been increasing in numerous fields, such as in medical applications (Yoneyama and Miyazaki, 2008), structures and composites (Lester et al., 2015), automotives (Jani et al., 2014), aerospace (Hartl and Lagoudas, 2007; Benafan et al., 2019), and even in robotics (Rodrigue et al., 2017).

**TABLE 1:** Comparison of actuators performance, inspired from Bhandari et al. (2012), Mohd Jani et al. (2014), Wang et al. (2018).

Actuator type	Stress (MPa)	Strain (%)	Efficiency (%)	Bandwidth (Hz)	Work density (J/cm^3^)
NiTi SMA	200	10	3	3	10
Piezoceramic	35	0.2	50	5,000	0.035
Single crystal piezoelectric	300	1.7	90	5,800	2.55
Human muscle	0.007–0.8	1–100	35	2–173	0.035
Hydraulic	20	50	80	4	5
Pneumatic	0.7	50	90	20	0.175
Ionic polymer-metal composites	0.3	40	30	0.1–100	0.0024
Dielectric electro-active polymer	2	100–380	60–90	1–10 k	3.4

**FIGURE 1:**
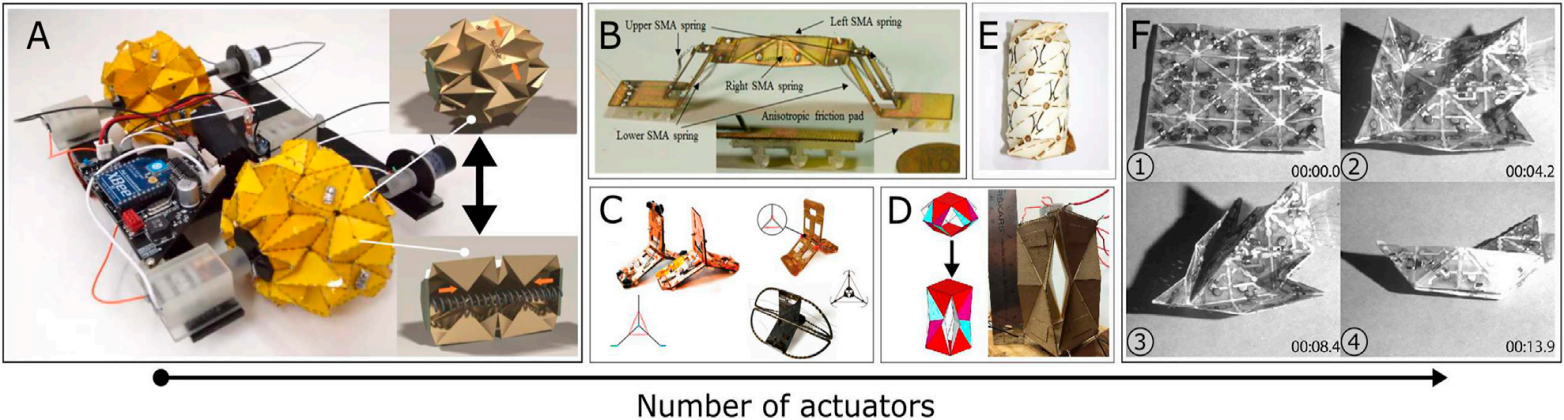
Diversity of SMA-Based origami robots. Origami robots arranged by the number of actuators: **(A)** A deformable wheel robot (Lee et al., 2013). **(B)** An inchworm-inspired crawling robot (Koh and Cho, 2013). **(C)** Different versions of a bi-modal locomotion origami robot of Paik’s group (Zhakypov et al., 2015; Zhakypov et al., 2017; Zhakypov and Paik, 2018). **(D)** A self-deployable lifting structure (Wood et al., 2016). **(E)** A “shape switching” module with controllable stiffness (Kim J. et al., 2015). **(F)** A “2D→3D” shape-morphing system (Hawkes et al., 2010).

Origami is a powerful method to introduce many unique and desirable structural properties such as auxetics, tunable stiffness, and multistability (Li et al., 2019; Kshad and Naguib, 2021). Robots inspired by folding mechanical structures known as “Origami robots” gained much attention recently. As for the robotics field, the introduction of origami engineering enables ‘semi-rigid’ properties. In other words, they exhibit the properties of both rigid and soft robots. For example, origami robots can be precise and support high loads like rigid robots but they can also be as dexterous and flexible as soft robots (Rus and Tolley, 2018). Moreover, the introduction of smart-material-based micro-actuators such as electro-active polymers (EAP) (Benouhiba et al., 2018) and SMA provides infinite possibilities to origami structures for self-deploying (Tolley et al., 2014; Wang et al., 2016) and dynamic shape-changing (Firouzeh and Paik, 2015; Kim et al., 2016). As shown in Figure 1, coupling with SMA makes origami robots achieve various tasks such as large ratio volume changing (Figure 1A), multi-type local motion (Figures 1B,C), high load-lifting (Figures 1D,E) and complex shape-morphing (Figure 1E). However, longitudinal motion-driven actuators (for example, tensile or bending behaviour-based actuation) are frequently investigated and commonly mentioned, when studies in SMA-based rotational motion actuation is still very limited in the literature.

Yuan et al. (2017b) presented a review on SMA-based rotary actuators. In this review, a classification based on actuators architecture and on a variety of supplementary mechanisms had been carried out. Stroud and Hartl (2020) presented a review on SMA-based torsional actuators in centimetre-size. Different aspects such as experimental characterisations and modeling relying on applications in the aerospace, biomedical, and automotive industries were well discussed. One needs to mention that the article focuses more narrowly on torsional actuators (for example, torsional tubes), and one is concerned here with rotational motion actuators for origami robots. As indicated by Peraza-Hernandez et al. (2014), the choice of actuators for self-folding structures (for example, a typical hinge-type fold’s “open-close” configurations requires ±π rad for self-folding), should be done according to criteria such as energy-density, simple geometry, and especially the actuators’ compactness since systems at reference configurations are often essential. Table 1 shows a summary of the performance of existing actuators in the literature. The reader is referred to the works by (Bhandari et al., 2012; Mohd Jani et al., 2014; Wang et al., 2018) for more details on actuator performance. Hines et al. (2016); Kim J. et al. (2019); Peraza-Hernandez et al. (2014) also review the families of actuators, presenting in particular the pros and cons of each family. Based on this comparison, SMAs can offer a trade-off between advantages such as significant strain, stress, high work density, and inconvenience such as relatively low energy efficiency and, low bandwidth. These properties make SMAs suitable candidates for origami robots’ actuation. In the present study, thermally activated SMAs for bidirectional rotational motion (BRM) of meso-scale robotics are considered. This type of actuator is capable of providing torque and angle in opposite directions for cyclic actuation using SME. This paper summaries the different research efforts related to SMA-based actuators for BRM, thus providing a survey and classification of current approaches and design tools that might be applied to them. Specifically, the remainder of this paper is organized as follows: Section 2 presents a brief summary of SMA materials and describes how they might be used for actuation. Section 3 presents the classification of SMA elements and a review of actuators’ architecture for BRM. Section 4 provides a discussion based on the review in terms of modeling, actuators’ characterization, performance prediction and designing, activation strategies, and sensing and controlling methods.

## 2 Brief Summary of Shape Memory Alloy

### 2.1 Shape Memory Alloys’ Thermal Mechanical Behavior

Shape memory alloys (SMAs) are a family of smart materials capable of sustaining large inelastic strains, depending on prior loading history, that can be recovered by heating or unloading. The composition of SMAs significantly influences their mechanical performance: the iron-based and copper-based SMAs are known as low cost candidates, but their applications are limited by their instability and poor thermomechanical performance (Lagoudas, 2008). The Ni-Ti based SMAs are the most conventional SMA materials and are much more applied by engineers than other SMAs, thanks to their outstanding mechanical properties.

**FIGURE 2:**
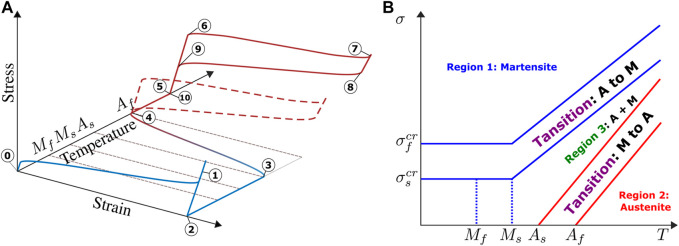
**(A)** An example of modeling of 1D stress-strain-temperature constitutive behavior of SMA. Shape memory effect: (0) to (4), pseudoelastic loop at constant high temperature: (5) to (10), the hysteretical behaviors are different according to the different temperatures. The figure is inspired by (Shaw, 2002). **(B)** An example of Brinson’s phase diagram, the figure is inspired by (Brinson, 1993).

**FIGURE 3:**
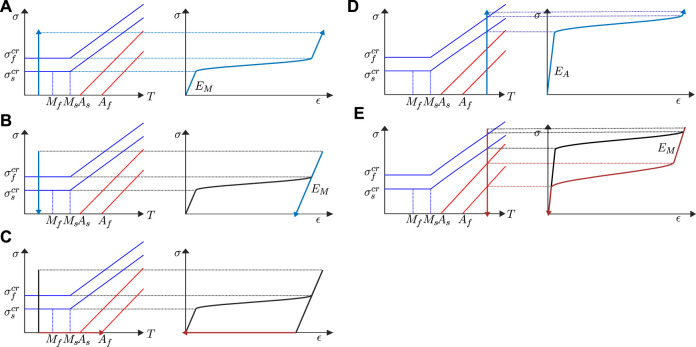
Examples of stress-strain relation of shape memory effect: **(A–C)** and of pseudoelastic behavior: **(D,E)**, the figure is inspired by (Saputo et al., 2020).

SMAs can exist in two distinct phases with three different crystal structures^3^ and therefore different properties (see Figure 2). The former is the high-temperature phase called austenite, and the latter is the low-temperature phase called martensite (Lagoudas, 2008). The difference in crystal structure between these two phases induces changes in the mechanical behavior. Furthermore, the core of the SME is constituted by a shear lattice dislocation due to thermal or magnetic active phase transformation. The thermally activated SMAs normally exhibit one-way shape memory effect (OWSME) (Mohd Jani et al., 2014), which is described as follow:

• Austenite converts to martensite (A→M) (see in Figure 3):
  1) The $A_{f}$ stands for the “austenite-finish-temperature,” which corresponds to the end of M→A transformation: the SMA is in a purely austenite state at this temperature. This state is known as the parent state or initial state of SMA.
  2) During a stress-free cooling process, the transformation starts to revert from austenite to martensite at $M_{s}$ “martensite-start-temperature” and finishes at $M_{f}$ “martensite-finish-temperature.” This process induces a formation of twinned martensite phase.
  3) When the twinned martensite is subjected to an applied stress larger than the “critic-start-stress” $\sigma_{s}^{\text{cr}}$,^4^ a reorientation process is initiated, resulting in growth of certain favorably oriented martensitic variants. The reorientation process is finished at the “critic-finish-stress” $\sigma_{f}^{\text{cr}}$ (see Figure 3A).
  4) After the elastic unloading, the detwinned martensitic state is retained with a residual strain $\epsilon_{L}$ (as described in Figure 3B).
• Martensite converts to austenite (M→A) (see Figure 3): when the SMA is heated above the “austenite-start-temperature” $A_{s}$ , the parent state begins to regain and the strain recovered due to the phase transformation from detwinned martensite to austenite is termed as the transformation strain $\epsilon^{t}$ (see Figure 3C).

Subsequent cooling to martensite will again result in the formation of self-accommodated twinned martensitic variants with no associated shape change, and the whole cycle of the OWSME can be repeated (Lagoudas, 2008). Mohd Jani categorized the shape change effect into three shape memory characteristics (Mohd Jani et al., 2014): 1) OWSME, 2) two-way shape memory effect (TWSME) that implies a tendency of the material to undergo mechanical strains with temperature cycling even when it is not pre-strained (Prahlad and Chopra, 2007), and 3) pseudoelasticity (PE)/Superelasticity. The OWSME-based actuators are usually more powerful, reliable and are widely implemented in many engineering applications (Mohd Jani et al., 2014).

### 2.2 Summary of Shape Memory Alloys’ Thermomechanical Models

This subsection briefly presents the various classes of models available in the literature for describing the behavior of SMAs. Unlike traditional materials, SMAs show high hysteresis during both M→A and A→M transformations. Physically, these hystereses are dissipation and assimilation of latent heat due to phase transformation that tends to slow down both heating and cooling processes (Velázquez et al., 2006). Consequently, in order to use SMAs in engineering applications, it is necessary to have an accurate understanding and description of their mechanical behavior.

#### 2.2.1 Model Classification

For years, various constitutive models have been developed by choosing different thermodynamic potentials, internal state variables, and their evolution equations, which provide various insights into the analysis and design of SMA actuators. For example, (Lagoudas, 2008), provides a comprehensive list of 1D and 3D constitutive models with different choices of thermodynamic potentials and internal state variables. As proposed by Cisse et al. (2016), the models presented here are classified into three categories: microscopic models, micro-macroscopic models, and phenomenological (macro) models.

1) The microscopic models are directly linked to initiation and evolution of multiple martensitic variants upon which superelasticity and shape memory effects are based (Paiva and Savi, 2006). Models in this category are intended to describe microstructural features in SMA behavior such as phase nucleation (Abeyaratne et al., 1994), interface motion (Duval et al., 2011), martensite twin growth, thus at the lattice or grain-crystal levels. They are useful to understand fundamental behavior occurring at the microscopic scale, but they are complex to apply at the device level.
2) Micro-macro approaches combine micromechanics and macroscopic continuum mechanics to derive constitutive laws of the material and often give good predictions. The development of micro-macro models requires the use of suitable observable variables and internal variables. The former usually consists in temperature T and external stress *σ* or strain *ϵ.* The latter usually comprises the volume fraction *ξ* of martensite and a mean transformation strain (Cisse et al., 2016). Fremond (1987) developed one of the earliest constitutive models for SMAs using internal variables. However, these models require very high computational cost, making them difficult to use for the design of engineering applications (Zhu et al., 2013).
3) Phenomenological models (Macroscopic models) are considered as a simplified version of micro-macro models. They describe average material behavior at macro-scale of SMA components using a limited number of internal variables (for example, only one internal variable like *ξ* ^5^ or two internal variables like $\xi_{T},\xi_{S}$ ^6^) by assuming the homogeneity and isotropy of material. In general, they are suitable to be used within numerical methods (such as the finite element method - FEM) in an efficient way to predict the effective behavior on the scale of millimeters to meters (Zhu et al., 2013; Elahinia, 2016).

The reader is referred to the works by Lagoudas (2008), Cisse et al. (2016) for more details on the models.

#### 2.2.2 Approaches on Phenomenological Models

The phenomenological models are the most popular models in literature compared to other approaches (Paiva and Savi, 2006), since they avoid the use of difficult-to-measure parameters such as free energy and use only clearly defined engineering material constants. Consequently, this approach plays an important role for SMAs-based engineering within SMAs’ behavior modeling context. In the literature, Lagoudas (2008) had implicated four critical aspects of characteristic modeling of SMA materials, such as: 1) the phase transformation kinematics, the hardening during phase transformations, and induced SME and PE behavior (see Figure 3); 2) the detwinning of martensite at low temperature, associated to the asymmetric response that SMAs exhibit in tension and compression; 3) the TWSME and the effects of reorientation; 4) the accumulation of plastic strains during cyclic loading. In this paper it is also claimed that the 1D constitutive model is acceptable for tensile and torsional applications such as SMA wires, rods, and tubes, and the statement has been validated experimentally by Prahlad and Chopra (2007), Chapman et al. (2011). Recent works on this topic frequently mention the 1D constitutive model developed by Liang and Roger (further for LRM) (Liang and Rogers, 1990) and by Brinson (further for MB) (Brinson, 1993). These approaches are based on the work of Tanaka (1986) that combines a mechanical and a kinetic law which governs the martensitic fraction of the material (Lobo et al., 2015). Sayyaadi et al. (2012) have compared these models with uni-axial tensile tests and concluded that these three models all agree well with their predictions of the PE of SMAs at high temperatures ($>A_{f}$). However, the models developed by Tanaka, and Liang and Rogers cannot be used for predicting the shape memory effect behavior with certain initial condition (for example, a residual strain due to a path including detwinning of pure martensite) (Sayyaadi et al., 2012). Besides the reduced-order model, 3D constitutive models using the FEM approach can be found in literature, “COMSOL” (Collet et al., 2009) and “ABAQUS” coupling with user subroutine (Peraza-Hernandez et al., 2013; Zhu et al., 2013) are often mentioned in studies. Although accurate and capable, due to the high hysteresis induced behavior and large strain during phase transition, finite element simulation can lead to a very high computational cost (Barbarino, 2015). However, they are mandatory when complex geometries are involved. Regarding the phenomenological models, additional details will be provided in Section 5.1, which is dedicated to practical applications.

## 3 Shape Memory Alloys-Based Actuators for Rotational Motion

**FIGURE 4:**
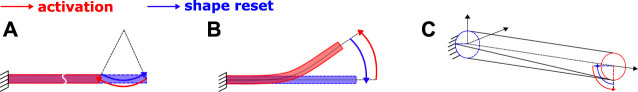
Examples of elementary SMA for uni-directional rotational motion: **(A)** Normal-motion-driven SMA element; **(B)** Bending-motion-driven SMA element; **(C)** Torsional-motion-driven SMA element.

SMAs made of NiTi have attracted wide interest in both research and industry due to crystal realignment, which is also the case for rotational motion actuators that are considered in this section, as they require both shape memory effect and high torque output. The performance of SMA actuators is primarily related to their metal composition and geometry. Huang mentioned that for SMA element designing, a trade-off should always be done (Huang, 1998): briefly, straight wires in tension offer small linear motion and high force; torsion bar and tube exhibit large rotation and small torque; cantilever strips for large displacement and small force; helical elements provide large linear motion and small force, or large rotation and small torque. It is worth reminding that the coil form design offers more design parameters than other simpler geometries. For example, for an SMA torsional coil spring actuator, a common category of actuator, the turns numbers, and diameter of a coil dictate its motion capacity, and the diameter of the wire relates to the output torque. A larger diameter leads to a higher output torque compared to the thinner one. However, beyond their linear actuation in spring or tendon forms, there is little variety research for actuation in torsional motion, which are useful for origami robots. SMA-based elementary actuators can be classified into three categories:

• SMA-based wires and linear springs (see Figure 4A): besides other forms of SMA-based actuators, wires are a more common form of actuators (Paik et al., 2010). However, their limited recoverable strain in the range of 4–8% requires them to have lengths up to 25 times longer than their intended stroke length (Rodrigue et al., 2017). A common way to increase output stroke without enlarging an actuator’s dimensions is to wind SMA wires around a cylindrical surface, resulting in a coil form actuator. The design consists in accumulating the normal deformation of the wire to global shear deformation of actuator in a single-coil form or double coil form (Kim S.-W. et al., 2015). In literature, novel arrangements and compliant mechanisms can be found to convert elementary linear strain to global torsional motion. For example, a tensile strain of two SMA wires in two sides of an elastic band can generate a global bending motion (Wang et al., 2008); an addition of torsional prestrain into the manufacturing process of SMA spring can offer an improvement of performance in terms of elongation rate and activation speed (Chung et al., 2019); an arrangement at constant and opposite eccentricity in a polymer matrix can convert the linear motion into a global twisting of the whole device (Rodrigue et al., 2015a). Rodrigue et al. (2017) had presented an overview of actuators and robots coupled with wire or linear spring elements. In this work, it is concluded that the optimization of actuators’ configuration for increasing their deformation range is the major challenge according to the structure size and the implementation of power source. As proposed by Paik et al. (2010), the actuators must be small enough to be embedded within the substrate material while sufficiently powerful to achieve folding. These characteristics make the integration of wire elements for self-folding structure a challenging engineering issue.
• SMA-based bending elements (see in Figure 4B): SMA bending actuators have been developed for robotics (Hawkes et al., 2010; Paik et al., 2010; Paik et al., 2012; Paik and Wood, 2012; Firouzeh and Paik, 2015; Zhakypov et al., 2016) and medical applications (Abadie et al., 2002; Abadie et al., 2009; Sheng and Desai, 2015; Sheng et al., 2017) for years, due to their small form factors, convenience in actuation, and compatibility with medical imaging. Large residual tensile and compression strains are locally distributed on the two sides of actuators in order to generate the rotational motion due to SME. Designs of bending-based actuators can globally be divided into two categories: 1) bending sheet: thin pre-annealed sheets are frequently mentioned in literature as an alternative to mechanical joints. The deformation occurs around an axis defined by the structure deformation rather than having a rotation between two separate elements, and there could also be some deformation of the structure surrounding the rotation axis (Peraza-Hernandez et al., 2014; Rodrigue et al., 2017). Hawkes et al. (2010) introduced a self-deployable origami structure using a universal crease with triangular module firm. 100 μmμm thickness pre-annealed sheet elements were integrated at folding creases. The prototypes were proven capable of realizing a “2D→3D” shape changing: a flat sheet maneuvers to fold toward complex shapes such as an airplane or a boat and maintain its shape using embedded magnets. However, the design is limited by the SMAs material properties. It was shown that deformation of a U-shaped metal sheet to its initial flatform is not possible (Firouzeh et al., 2013), because of the plastic deformation of the material that forms a curvature at the hinge. A solution consists in introducing extra curvature to achieve a larger rotation angle. Consequently, additional machining processes such as laser cutting and SMAs layer’s pre-annealing are necessary (Paik et al., 2010; Zhakypov et al., 2016). 2) bending wire or cantilever: single or group of linear and arc-shaped SMA wires that are arranged to provide bending motion (Shin et al., 2016). Investigations on SMA-based bending beams and cantilever are carried out for applications that require larger output torque (Abadie et al., 2009; Wang et al., 2016).
• SMA-based torsional elements (see Figure 4C): since the conversion between linear motion and rotational motion requires supplementary mechanisms, resulting in energy dissipation (friction, extra mass) and operation space requirement, studies on SMA-based torsional elements have been developed in the last decade. Using this type of design, large recoverable shear deformation^7^ can be simply obtained by gripping both ends without any extra mechanism. Consequently, the fabrication and assembly process can be simplified. Different designs of elementary torsional actuators can be found in literature, rotary actuation capability are experimentally proven using torsional strips (Tobushi et al., 2008; Tobushi et al., 2010; Tobushi et al., 2013), torsional bands (Shim et al., 2015), torsional wires (Kim J. et al., 2015) and torsional tubes (Benafan et al., 2019). Besides the shear-strain-driven-actuators, investigations on torsional coil spring (Salerno et al., 2013; Sheng and Desai, 2015; Sheng et al., 2017) that convert the local normal strain to global shear strain are carried out and results show an improvement of motion range and reduction of output torque compared to the wire form torsional actuator. It is worth reminding the works introduced by Kim J. et al. (2015) and by Wood et al. (2016), that presented the origami-based self-deployable structure coupled with torsional wire elements. The former was based on a “Kresling” pattern, and the latter was based on a “Waterbomb” pattern. Prototypes showed a global shape morphing driven by torsional actuation at folding creases with more than 90° rotation. Additionally, interesting structural performance such as “buckling effect” due to pattern design were observed Kim J. et al. (2015). However, these structures required external force to be re-folded after activation.

To sum up, normal-strain-driven actuators offer different advantages such as simplicity of manufacturing, simplicity of multi-physical modeling, and suitability for conventional cooling. However, the requirement of operational space and complexity of supplementary mechanism limits the capacity of this design. Bending-elements offer the same level of motion and allow the possibility of size reduction of actuators but require supplementary fabrication. Torsional elements have shown an interesting potential due to their larger angular motion-range and larger output torque within a limited space. Regarding origami-inspired structures, which require large torsional actuation within very limited operational space (Hawkes et al., 2010) at folding creases, these characteristics makes them appropriate candidates for origami-inspired robotics (Koh et al., 2014; Kim J. et al., 2015).

## 4 Actuators for Bi-Directional Rotational Motion

**FIGURE 5:**
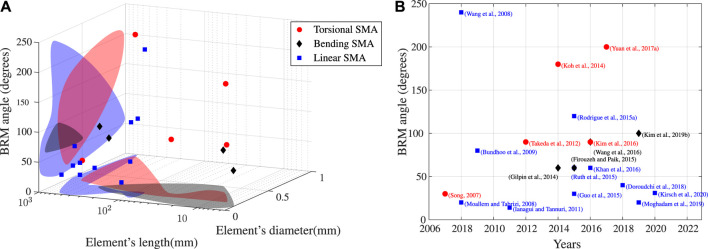
Data report of performance of SMA-based BRM actuators from literature: **(A)** shows the relationship between the elements dimension and bi-directional motion range of actuators. Colors corresponds to actuator types. Surrounding colors show the boundaries of existing prototypes: For given angle, L-SMA require larger values in terms of SMA lengths; according to the same diameter/thickness of SMAs, T-SMAs offer larger angles than B-SMAs. Publishing-time according to the presented works is provided on the **(B)**.

Conventional SMAs can only achieve unidirectional actuation. Consequently, it is necessary to provide an external force to carry out a repeatable bi-directional motion. According to the nature of this force, two classes of SMA actuators can be defined: passive bias type SMAs actuator and active bias type SMAs actuator (Georges et al., 2013). The former one, composed of an SMA element and a bias load (constant mass, linear or nonlinear stiffness spring), offers the simplicity of modeling and manufacturing but its performance (dynamic response and motion range) is highly dependent on the choice of passive element. The latter one, which consists of two antagonistic SMA elements, has faster speed of response than the former one, but it requires more power (Khan et al., 2016) and the output torque is restricted by the stiffness of the antagonistic components. These two categories are described in the following. Figure 5 and Table 2 provide an overview of the principles, dimensions, performance, heating and cooling strategies, models used for design and controlling issues for a large number of SMA-based BRM actuators.

**TABLE 2:** Summary of existing SMA-based actuation system for BRM.

Elementary SMA type^a^	Dimension^b^		Performance^c^		Heating and cooling^d^		Model^e^	Sensing and controlling^f^		
	D	L	MR	OT				CM^g^	TS^h^	MS^i^
L-SMA Liu et al. (2019)	—	80	90	—	JH	NAC	LRM/BM	MOSFET	TM	VA
L-SMA Khan et al. (2016)	0.076	85	60	—	JH	NAC	—	PID	—	RE
T-SMA Basaeri et al. (2014)	0.254	—	20	—	JH	NAC	LRM/BM	—	—	RE
T-SMA Redmond et al. (2010)	0.038	—	20	—	JH	NAC	—	—	—	RE
T-SMA Song (2007)	0.38	736	30	—	JH	FAC	—	PID	—	RE
T-SMA Takeda et al. (2012)	0.25	40	90	4	EH	NAC	EXP-driven	—	—	—
T-SMA Yuan et al. (2017a)	1	753	200	—	JH	—	—	—	—	IP
L-SMA Bundhoo et al. (2009)	0.035	385	80	2	JH	NAC	LRM/BM	PWM-PID	TC	RE
L-SMA Kirsch et al. (2020)	0.025	550	31	—	JH	NAC	—	—	—	RE
L-SMA Guo et al. (2015)	0.25	370	30	—	JH	NAC	LRM/BM	PI	—	RE
L-SMA Ruth et al. (2015)	1	700	60	1.4	JH	NAC	EXP-driven	SMC	—	—
L-SMA Doroudchi et al. (2018)	0.2	500	40	—	JH	NAC	LRM/BM	Open-loop	—	RE
L-SMA Ianagui and Tannuri (2011)	0.2	150	14	2	JH	PEC	IM	Close-loop	—	RE
L-SMA Moghadam et al. (2019)	0.2	500	20	20	JH	NAC	LRM/BM	SMC		RE
L-SMA Nespoli et al. (2010)	0.2	—	160	0.1	JH	NAC	—	—	—	—
L-SMA Moallem and Tabrizi (2008)	0.38	964.5	20	15	JH	NAC	LRM/BM	PID		RE
L-SMA Lan et al. (2009)	0.125	—	14	—	JH	NAC	—	PID	—	RE
L-SMA De Sars et al. (2010)	0.22	—	10	17	JH	NAC	FEM	SMC	—	—
L-SMA Rodrigue et al. (2015a)	0.15	100	120	—	JH	—	LRM/BM	—	—	IP
B-SMA Firouzeh and Paik (2015)	0.1	4.5	60	0.83	EH	NAC	EXP-driven	Close-loop	IR camera	BS
B-SMA Gilpin et al. (2014)	0.51	17	60	0.94	JH	HS	—	Close-loop	—	AM
B-SMA Abadie et al. (2009)	0.2	—	50	0.37	TEH	PEC	RLM	—	TC	VA
B-SMA Kuribayashi (1989)	0.05	—	80	0.04	—	NAC	EXP-driven	—	—	—
B-SMA Kim et al. (2019b)	0.25	320	100	15	JH	NAC	EXP-driven	PID	IR camera	RE
B-SMA Wang et al. (2016)	0.15	190	90	—	JH	—	—	—	—	IP
L-SMA Wang et al. (2008)	0.2	76	240	—	JH	NLC	—	—	—	IP
T-SMA Sheng et al. (2017)	0.5	—	40	10	JH	NAC	LRM/BM	PI	TM	VA
T-SMA Koh et al. (2014)	0.4	12	180	—	JH	NAC	LRM/BM	—	IR camera	IP
T-SMA Kim et al. (2016)	0.25	8	90	—	JH	NAC	LRM/BM	—	—	IP

a L-SMA, B-SMA, T-SMA: SMA wire or linear spring elements, SMA Bending elements, SMA torsional element.

b D, L (mm): SMA element’s diameter or thickness, element’s length.

c MR, OT: motion range (degree), output torque (mNm) for BRM actuation system.

d JH, EH, TEH, FAC, NAC, NLC, HS, PEC: Joule heating, external heater, Thompson effect heating, forced air convection, natural air convection, natural liquid convection, heat skin, Peltier effect cooling.

e LRM/BM, RLM, IM, FEM, EXP-driven: Liang and Roger or Brinson model, Raniecki and Lexcellent model, Ikuta model, finite element model, model driven by experimental data.

f CM, TS, MS: controlling methods, temperature sensing methods, motion sensing methods.

g PID, SMC: proportional–integral–derivative controller, sliding mode controller.

h TC, TM: thermocouple, thermomister.

i RE, VA, IP, BS, AM: rotary encoder, video acquisition, image processing, micro-bending-sensor, accelerometer.

### 4.1 Passive Biased Actuator

As reminded before, an SMA-based actuator should first be pre-deformed at low temperature ($<M_{s}$) and then activated to a higher temperature ($>A_{s}$) to initiate its controlled contraction due to the M→A transformation. This controlled contraction can then be used to generate mechanical work.

**FIGURE 6:**
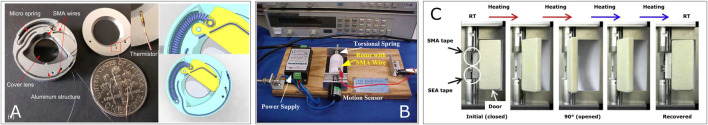
Examples of BRM actuator using SMA wire element: **(A)** A mesoscale SMA actuator for cleaning the contaminated lenses of surgical cameras during minimally invasive robotic surgery (Liu et al., 2019); **(B)** A rotary servo driven by a coil form SMA wire, the shape “re-set” torque is provided by a torsional spring (Song, 2007); **(C)** An opening and closing door device capable of a two-way rotary motions using a combination of a SMA and SEA (superelastic alloy). The strip was biased passively by a PE element for position resetting (Tobushi et al., 2013).

Passive elastic elements have been widely used in SMA-coupled innovations since they are simple to be engineered and integrated into structures. Although, beyond the utilization of traditional springs, elastic or super-elastic elements can take the place of springs to achieve specific requirements, such as reducing working space or amplifying rotation stroke. However, the spring force is passive and decreases as the pulley moves back to the original position. Without high initial tension, residual displacement is unavoidable (Lan et al., 2009). Jenkins and Landis (1995) presented an SMA-actuated rotating arm for moving the cover glass of a solar cell on NASA’s Mars Pathfinder using a wire actuator with 0.15 mm diameter activated with Joule-effect heating. A similar design at a miniature scale has been implemented by Liu et al. (2019) (see Figure 6A) for medical applications, by Khan et al. (2016) for robotics applications and Basaeri et al. (2014) for aeronautic applications. Design with coil form torsional SMAs with passive spring are studied in Lucy and Center. (1996), Redmond et al. (2010), Song (2007) (see Figure 6B); shear strain-driven actuators are presented in Tobushi et al. (2010), Takeda et al. (2012), Prahlad and Chopra (2007), Chapman et al. (2011). Besides utilizing conventional springs with linear stiffness, some authors introduced functional materials such as pseudo-elastic SMA or composite (Kim et al., 2002) to improve the actuators’ performance while resetting the actuator’s pre-strained state. For example, Yuan et al. (2017a) used a helical spire form rotary actuator driven by a linear SMA wire. The ‘re-arming’ force generated by structural stiffness is made by Acrylonitrile Butadiene Styrene (ABS). Song et al. (2016) presented an autonomous swimming robot using flipper actuators which consist of four SMA wires with 0.15 mm in diameter and anisotropic materials (Kim et al., 2012; Ahn et al., 2012) with three types of scaffold structure (oriented ABS scaffolds in PDMS matrix). The prototype has been proven to mimic the continuous deformation (bending and torsion) of the forelimb of a sea turtle. Takeda et al. (2012) presented two-way rotary motions of an opening and closing door device (see Figures 6A,C solar-powered active blind model driven by a SME-based twisting strip. The strip was biased passively by a PE behavior element for to reset position. The rotation modular was shown capable of rotating of 45° with fatigue life longer than $3\times 10^{6}$ cycles. One needs to remind that these constant stiffness elements are frequently used to characterize SMA’s performance (Prahlad and Chopra, 2007; Chapman et al., 2011; An et al., 2012), which somehow constitutes a passive biased actuator.

### 4.2 Active Biased Actuator

The passive bias components discussed above require fine tuning to cooperate correctly with the actuator. Instead of using passive bias elements to “re-arm” the actuator, integrating active bias elements such as an antagonistic SMA element is a very efficient way to create devices capable of producing differential motion paths and two-way motion (Georges et al., 2012). The current rotary actuators using this design can be classified based on their actuation elements, which we have previously described in Section 3.

#### 4.2.1 Linear Shape Memory Alloy Element With Mechanical Joint

This design consists in converting the linear contraction strain of the SMA element to rotational motion using a mechanical articulation such as a pulley or a compliant joint, thus creating a torque about this joint on contraction, where the torque arm is the distance between the SMA wire and center point of the joint (Rodrigue et al., 2017). Consequently, design parameters are the structure geometry and also the dimension of the SMA wire. For example, in their work, Kirsch et al. (2020) shown an actuation system consisted of an antagonist wire element of 0.025 mm diameter and 550 mm length and a pulley of 3 mm in diameter. The SMAs are heated using the Joule effect and cooled using natural air convection, resulting in a rotation stroke of 31° with an actuation frequency of 10 Hz.

**FIGURE 7:**
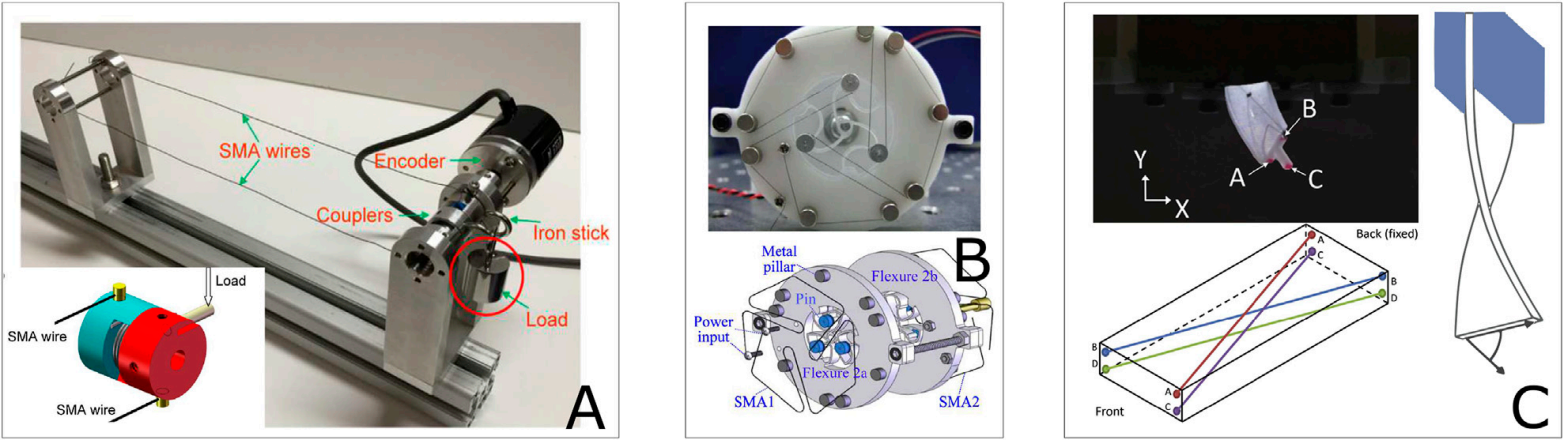
Examples of BRM actuator using SMA wire element: **(A)** A rotary modular using “antagonistic Linear SMA + pulley” design with a intermediary torsional spring (Guo et al., 2015); **(B)** A rotary modular using “antagonistic Linear SMA + optimized spring-slack element + pulley” design with compact arrangement of linear SMA (Lan et al., 2009); **(C)** A BRM actuator consists of four SMA wires embedded in a soft matrix. The actuator is capable of performing banding and twisting motion in opposite directions (Rodrigue et al., 2015b).

Doroudchi et al. (2018) used the same design and a similar length for the SMAs but a 20 mm diameter pulley. The actuator provides only 1° of stroke with the same frequency, using only forced air convection. Based on this typical design, various contributions can be found in literature: 1) supplementary mechanism or complex design to amplify the rotary stroke; 2) advanced controlling model for the accuracy of rotary system; 3) enhanced heating or cooling method for improvement of response dynamic; Focusing on the first improvement, Guo et al. (2013), Guo et al. (2015) introduced a bi-directional rotary actuator with a torsional intermediary compliant spring, and the stroke was amplified from 10 to 30° (see Figure 7A). Bundhoo et al. (2009), Gilardi et al. (2010) developed a tendon-driven actuation system for artificial fingers with a complicated feedback control system, the antagonist SMAs are both embedded with a spring and a stopper to amplify the stroke up to 80°. Based on this approach, Lan et al. (2009) developed a BRM actuator equipped with a pair of SMA wires with a compact arrangement and an optimized structural contractive part (see Figure 7B). The actuator has proven ability of a 14° angular stroke. Nespoli et al. (2010) introduced a meso-scale rotary modular using two wire elements. The wire elements are pre-annealed into flat spring to achieve a compact design, the prototype shown its capability of offering a rotation stroke of 160° corresponding to 4.5 mm displacement in opposite directions. Concerning the second improvement, Moallem and Tabrizi (2008) introduced a detailed multi-physics 1D model, which firstly used a LRM based differential form martensite factor. The BRM actuation prototype has be proven capable of 20° stroke rotation with a maximum error of 0.5° using motion feedback. Based on this approach, Moghadam et al. (2019) showed that the introduction of BM and a cascade control offers better accuracy than the former. Ruth et al. (2015) proposed a self-sensing actuator using electrical resistance measurement, and the prototype showed a result of a 30° stroke with a 2° error. For the third improvement, the work of Doroudchi et al. (2018) used forced air convection to accelerate the cooling time, resulting in 5 Hz with a 4° stroke and 10 Hz with a 1° stroke. Another method is presented by Ianagui and Tannuri (2011). In this work, a Seeback effect-based (Romano and Tannuri, 2009) cooling tablets was introduced. The prototype has been proven capable of providing ±7° motion range at a frequency of 1.3 Hz. Beyond the utilization of conventional design, novel architecture of actuators and arrangements provide more possibilities for applications. For example, Rodrigue et al. (2015b) shown an actuator of a rectangular polydimethylsiloxane (PDMS) matrix embedded with two pairs of SMA wires maintaining a constant eccentricity from the middle plane across the thickness (see Figure 7C). The prototypes have been proven to offer both bending and rotating motion using Joule effect heating. Koh and Cho (2013) have introduced an inchworm-inspired origami-based climbing and steering robot. The steering motion was induced by SMA springs in the body. The antagonistic SMA springs-driven flexible hinges in the leg with anisotropic friction pads provided the climbing motion. Zhakypov et al. (2015) presented a low profile centimeter-scale origami robot actuated by three antagonistic SMA linear springs. The 4 g weight robot is capable of jumping as high as five times its height. De Sars et al. (2010) developed a multi DOFs structure actuated by thin waveform NiTi layer springs mounted in an antagonist configuration and directly integrated into the structure of an endoscope.

#### 4.2.2 Shape Memory Alloy-Based Flexural Joint

Besides the linear SMA, bending-strain-driven SMA-based actuators are also mentioned in literature. This design is an alternative to mechanical joints. Instead of transforming linear motion to rotation, a hinge-like joints using reversible bending material at the rotation axes is carried out in order to achieve the bi-directional actuation. In literature, this kind of design is usually referred to as “flexural joint” (Peraza-Hernandez et al., 2014; Rodrigue et al., 2017). Paik’s and coworkers presented several researches on Origami robot coupling with SMA-based actuation in bending layer form (Paik et al., 2010; Paik et al., 2011; Paik et al., 2012; Paik and Wood, 2012; Firouzeh and Paik, 2015; Zhakypov et al., 2016). The pre-annealed thin sheets form SMAs are generally mentioned as the elementary actuators. Based on this design, different contributions were carried out: the actuator was first presented in 2010 (Paik et al., 2010) using a sheet of 500 μmμm in thickness with fabrication process, an analysis of the designing of external heater was also addressed, and an experimental test bench is introduced. The actuator was demonstrated capable of rotating with 180°. A “print-on” stretchable electrical circuit was then studied to achieve the sensing and controlling of this miniature self-folding based actuator (Paik et al., 2011).

**FIGURE 8:**
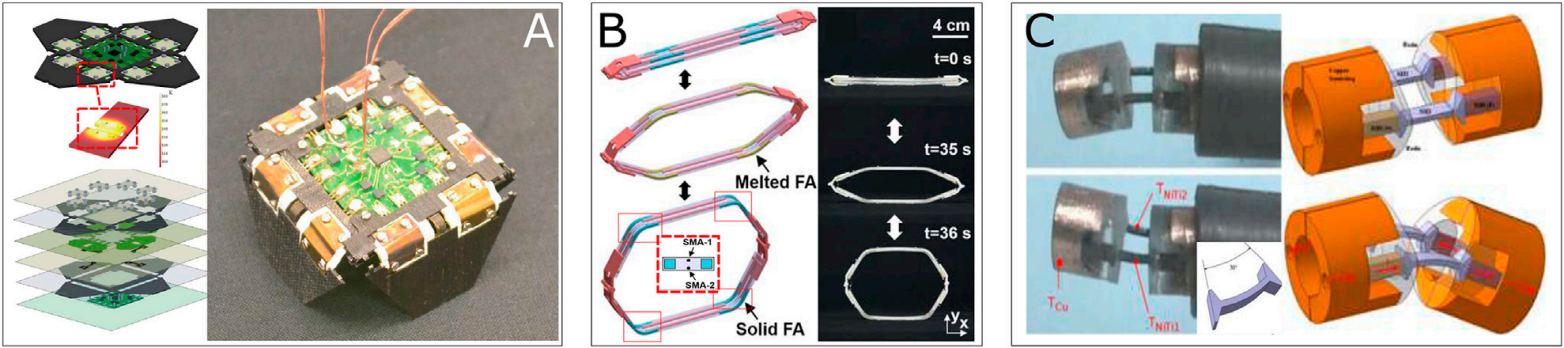
Examples of BRM actuator using SMA bending element: **(A)** A prototype of “Robogamis,” a two-dof local motion robot using four pairs of thin-layer-form antagonistic SMA (Firouzeh and Paik, 2015); **(B)** A self-deployable structure with four hinge-like joints for BRM, four pairs of antagonistic thin-bending-rod form SMAs have been embedded (Wang et al., 2016); **(C)** A prototype of rotational micro-actuator for active endoscopy application with a pair of antagonistic bending cantilever form SMAs. A Peltier-effect-based heater and cooler has been integrated (Abadie et al., 2009).

The design was further improved using a novel arrangement (Paik and Wood, 2012), an “S” form pre-annealed sheet actuator coupled with a Joule effect-based flexible heater was presented. The results have shown a capability of a 180° rotation in opposite directions. Zhakypov et al. presented a complete study including designing based on the LRM, characterizing, and angle controlling of actuator (Zhakypov et al., 2016). The actuator was further embedded with 1 SMA spring with two flexible articulations in the middle to realize a “clamp—jump” motion (Zhakypov and Paik, 2018). Firouzeh and Paik (2015) presented a four-legged origami robot named “Robogami Crawler” that is capable of providing locomotion with 2 dofs using four folding modules. Each module consists of a pair of flat 2D SMA with 100 μmμm thickness sheets in antagonist configuration (see Figure 8A). Elementary SMA was proven to rotate with a maximum angle of 120° with 5–16 mN m^8^ output torque. The antagonist pair is proven to provide a ±60° motion while providing 0.83 mNm torque to overcome the friction. Apart from works of Paik’s group, Gilpin et al. (2014) developed a Hexroller shape close chain combined with a series of personalized bending SMA layers is developed in order to realize locomotion in one dimension. Abadie et al. (2002) presented a micro-actuator called “Ω modular” with a Ω form bending strip. One needs to highlight that he first used the Peltier effect and Thompson effect to accelerate the thermal exchanging process. Abadie et al. (2009) further enhanced this design with a pair of bending cantilever and a thermoelectric temperature controller for active endoscopy applications (see Figure 8C). Beyond the sheet form geometry, other conceptions such as bending wires, bending strips, and bending cantilevers have been investigated. Since the maximum strain of SMA for cyclic applications is limited to 3–4%, single bending elements usually offer relatively lower strokes than other designs. Thus the actuation system often arranges the bidirectional SMA elements in series or in parallel to achieve either a larger stroke or a higher output torque globally. Kuribayashi (1989) presented a millimeter-sized actuator using two pairs of thin bending SMA strips. The prototype can provide a stroke of ±80° with an output torque of 0.04 mNm. Wang et al. (2016) introduced a “hinge-like” BRM actuation modular consisted of 0.15 mm diameter and 15 mm actuation length (190 mm of total length) antagonist bending wires embedded into a PDMS elastomer matrix. The prototype was proven capable of providing ±90° rotation and of blocking its shape^9^ using a thermal-controllable stiffness joint made of fusible metal^10^ (see Figure 8B). Kim Y. et al. (2019) developed an actuator to provide angular displacements in both clockwise and counter-clockwize directions with compliance using 28 pairs of bending wires in parallel in opposing way. Roudaut et al. (2013) proposed novel display conceptions for mobile devices that can morph their shape using SMA actuation driven by an SMA composite with a mesh of bending SMA wire. Wang et al. (2008) developed an embedded SMA wire actuated biomimetic fin for underwater propulsion. A micro-robot fish (146 mm in length, 30 g in weight) using antagonistic SMA wire with a silicon-based elastic substrate is shown as a prototype. This actuator can achieve an angle of ±90°.

#### 4.2.3 Shape Memory Alloy-Based Torsional Joint

The twisting deformation-based elements offer several benefits, such as design simplicity and large shear strain. However, comparing other forms of actuators, the research on bi-directional actuation using twisting elements is very limited.

**FIGURE 9:**
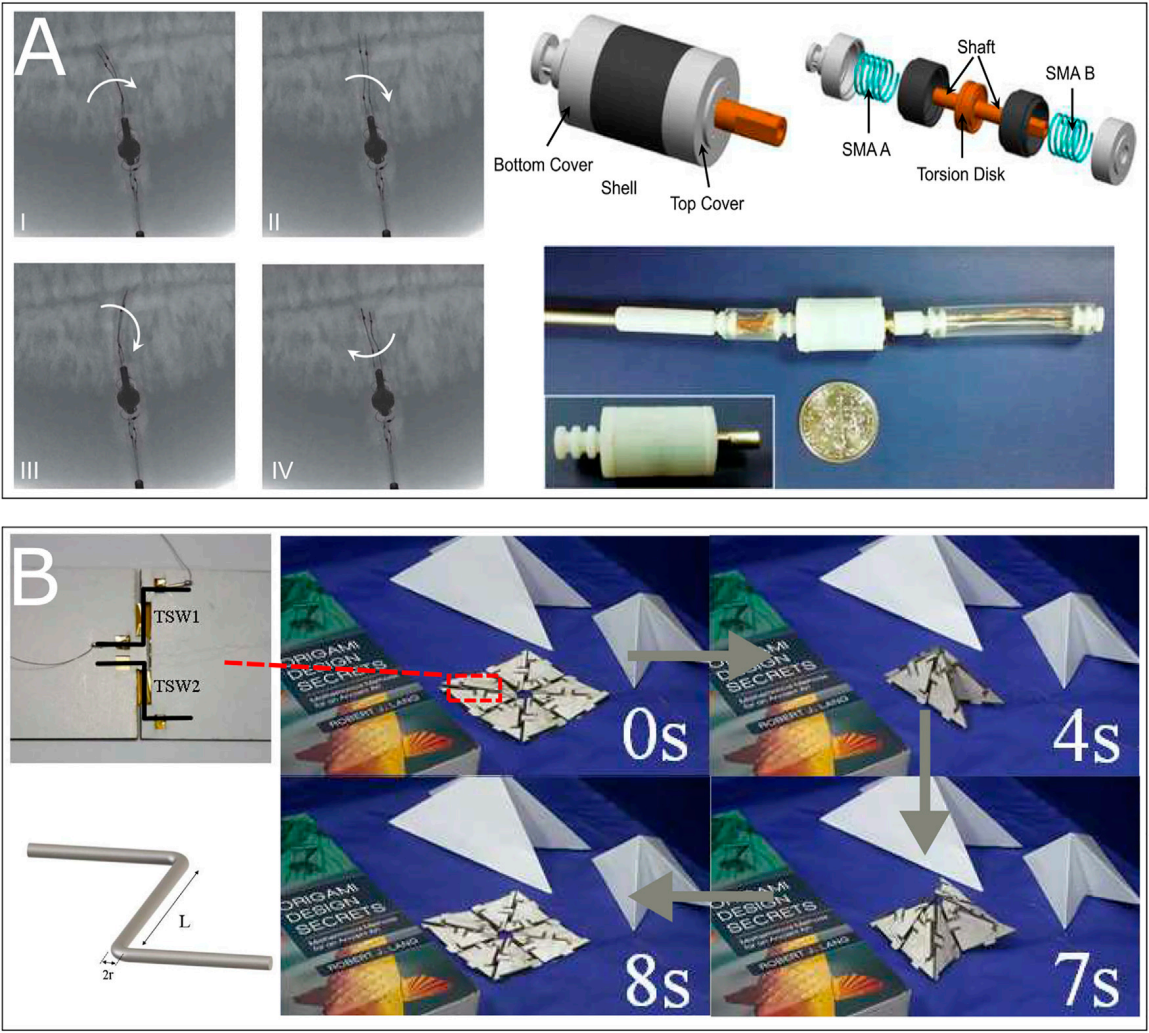
Examples of BRM actuator using SMA torsional element: **(A)** A meso-scale BRM modular embedded 2 antagonist torsional springs for surgical application (Sheng et al., 2017); **(B)** A meso-scale self-deployable paper-based open origami structure that capable of switching between 2D-3D state (Koh et al., 2014).

Sheng and Desai (2015), Sheng et al. (2017) introduced a torsional actuator using torsional springs in antagonist configuration for a surgical robot prototype to demonstrate its working performance in humid environment under C-Arm CT image guidance. The study shown a rotation stroke of ±20° at 0.05 Hz (see Figure 9A). Koh et al. (2014) introduced a torsional SMA wire actuator embedded in patterned origami structures. The wires of 0.4 mm in diameter and 12 mm in length were pre-twisted at 360° in the opposite directions for each and resulting in achieving a typical origami 3D-flat transition within 4s using an applied current of 0.8 A (see Figure 9B). Based on this approach, Kim et al. (2016) presented a shape-shifting system consists of distributed self-deployable origami modules using a modified bi-stable “Kresling” pattern. Two pairs of antagonist 250 μmμm diameter and 8 mm length torsional wires were embedded on each module. The prototype was proven capable of switching the shape driven by 90° BRM actuation at folding creases with 1A for 5 s. One needs to highlight that the system was capable of shape-blocking thanks to structural bi-stability due to the origami pattern. Similar design of actuation system in a larger scale was investigated in the eronautic domain, Benafan et al. (2019) developed a bi-directional torsion actuator using antagonist SMA tube for NASA’s spanwise adaptive wing (SAW) project. The SMA tube has 3 mm thickness and 304 mm in length, resulting by a folding motion of ±70° of wings during a flight test.

## 5 Discussion

This section introduces a discussion on critical topics related to SMA actuators. The characteristics discussed here are the available analytical tools for describing actuators’ behavior, performance prediction and designing driven by inverse model, the temperature controlling strategies, sensing, and position/force control.

### 5.1 Analytical Tools for Bi-Directional Shape Memory Alloy Actuators

SMAs’ high hysterical behavior usually depends on their temperature history and applied stress. Consequently, a reliable model becomes necessary for the actuator design. The discussion about bi-directional SMA actuators is divided in two parts: 1) thermal modeling for the computation of the temperature distribution; 2) thermomechanical modeling for prediction of the motion range and actuation torque.

#### 5.1.1 Electro-Thermal Model

Since the SMAs mechanical behavior depends on the thermal evolution path, it is necessary to describe the temperature distribution of SMAs. For most of bi-directional actuation, the geometry of SMA element is usually straightforward. Hence 1D thermal balance modeling can be used by assuming the uniformity of temperature distribution *T* with Biot condition *B* (Shahin et al., 1994), such as:

$$\rho V\left(C\frac{dT}{dt}+\text{Δ}H\frac{d\xi }{dt}\right)=\phi_{s}-\phi_{\text{dissipation}}=I^{2}R-hS\left(T-T_{amb}\right)$$ (1)

$$B=\frac{hl}{\lambda }<0.01$$ (2)

where $\phi_{s},\phi_{\text{dissipation}},\text{Δ}H,C,h,\rho ,\lambda ,S,V,l,\xi ,I,R,T_{amb}$ the thermal source flux, the thermal dissipation flux, latent heat rely on the phase transition of SMA, the thermal capacity, the convective heat transfer coefficient, the actuator’s density, the thermal conductivity density, the surface area of the SMA wire of actuator, the actuator’s volume, activation length, the martensite factor, the input current, the electrical resistance and the temperature of the surroundings, respectively. The equation corresponds to the typical case (see Table 2) with Joule effect heating and natural air convection which is a very common use of SMAs-based actuators. The partial differential equation can be implemented easily in order to realize a real-time modeling of actuator thermal behavior. Moreover, Abadie’s model (Abadie, 2000) utilized the same method, but the differential element corresponds to the Peltier effect, and Paik and Wood (2012) have used the 3D FEM approach to predict the temperature distribution for cantilever or thin sheet elements. Additionally, the dissipation term can be modified to meet different cooling methods, such as forced air convection (Doroudchi et al., 2018), water or oil-based convection (Wang et al., 2008) and thermal conduction or thermo-electrical effect based dissipation method (Shahin et al., 1994; Abadie, 2000; Romano and Tannuri, 2009). In literature, investigation on the change due to thermal convection coefficient and electrical resistance are carried out to improve the models’ performance. Shahin et al. (1994) introduced a complex formulation to estimate the convection coefficient of air, and he offered a simpler expression based on a horizontal cylinder in free air under gravity:

$$h\left(T\right)=-0.379+20.563{\text{log}}_{10}\left(T\right)$$ (3)

One needs to remind that for a certain case like thin linear SMAs wires, the variation of electrical resistance of actuator can show a nonlinear behavior due to phase transition and shape-changing (Ruth et al., 2015). Such investigation is presented in Velázquez et al. (2006), in which he developed a temperature and deformation-based function of electrical resistance, resulting in improvement of modeling precision. The influence of latent heat is another factor that has been usually neglected for mathematical simplicity (Moallem and Tabrizi, 2008; Peraza-Hernandez et al., 2014; Guo et al., 2015; Liu et al., 2019; Moghadam et al., 2019). Besides the utilization of the reduced-order model, the numerical approach using 3D FEM method are presented in the literature in order to study the temperature evolution more accurately (Abadie et al., 2009; Paik and Wood, 2012; Firouzeh and Paik, 2015). For example, Paik and Wood (2012) shown a local heating effect due to personalized heaters to optimize energy efficiency using FEM. However, these models provided only the prediction of thermal behavior without coupling mechanical hysteresis of SMAs.

#### 5.1.2 Thermomechanical Modeling of Actuator for BRM

**FIGURE 10:**
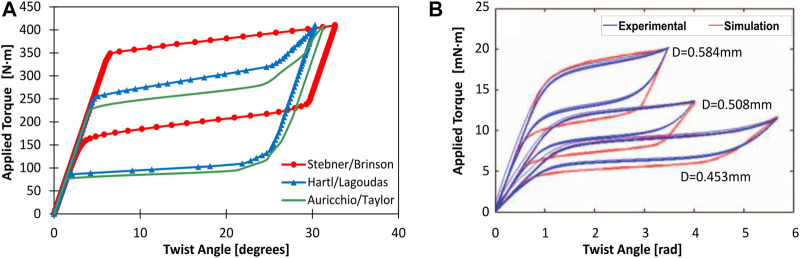
An example of simulation results with 3D FEM frameworks for pure torsional SMA elements: **(A)** Torsional PE behavior of a centimetre-size SMA tube form actuator for different models (Abaqus) (Zhu et al., 2013). **(B)** Torsional PE behavior of millimetre-size SMA wire form actuators for different diameters (Abaqus), the figure is reproduced from Chapman et al. (2011).

1D quasistatic phenomenological models implemented in numerical software are frequently utilized to predict the thermomechanical response of actuators. The LRM/MB are demonstrated as a suitable model for prediction of the quasistatic and dynamic response of passive or active biased normal stress actuator for BRM (Gilardi et al., 2010; Doroudchi et al., 2018; Liu et al., 2019; Moghadam et al., 2019). Moallem and Tabrizi (2008) introduced the rate of martensite factor evolution based on LRM for dynamic response prediction. Based on this approach, Moghadam et al. (2019) shown an improvement of precision of controlling using a modified version of MB. Additionally, the LRM model is validated experimentally using antagonistic SMA torsional spring in (Sheng et al., 2017). It is worth reminding that Liang and Roger’s hardening function are given as,

$$\begin{array}{cc} M\;\to \;A: & \xi =\frac{\xi_{M}}{2}\left\{\text{cos}\left[\frac{\pi }{A_{f}-A_{s}}\left(T-A_{s}-\frac{\sigma }{C_{A}}\right)\right]+1\right\}\\ & \text{for }A_{s}+\frac{\sigma }{C_{A}}\leq T\leq A_{f}+\frac{\sigma }{C_{A}}\\ A\;\to \;M: & \xi =\frac{1-\xi_{A}}{2}\text{cos}\left[\frac{\pi }{M_{s}-M_{s}}\left(T-M_{f}-\frac{\sigma }{C_{M}}\right)\right]+\frac{1+\xi_{A}}{2}\\ & \text{for }M_{s}+\frac{\sigma }{C_{M}}\leq T\leq M_{f}+\frac{\sigma }{C_{M}} \end{array}$$ (4)

with $\xi_{M},\xi_{A},C_{A},C_{M}$ the initial values of martensite factor for each transformation process, the material constants that indicate the influence of stress on the transition temperatures, respectively (Liang and Rogers, 1990). A simpler formulation is frequently mentioned in literature concluded that it is acceptable for motion prediction (Shahin et al., 1994; Zhakypov et al., 2016; Rodrigue et al., 2017; Liu et al., 2019) by replacing the initial value $\xi_{M}$ and $\xi_{A}$ by a scalar value 1, that assuming every actuation cycle begin with a 100% phase transition. Facing others forms of actuator, the capability of the reduced-order model is proven according to certain designs. Based on the study of Zhakypov et al. (2016), the LRM is proven acceptable for pre-annealed thin SMA sheet with a curvature by assuming pure bending since the curvature length is much larger than the layer’s thickness. The Lexcellent 1D model was implemented in Abadie et al. (2002), and Paik and Wood (2012) using a stack of thin layers assumption for bending cantilever and bending sheets form actuators. However, only tensile behavior was considered in these models. For shear-stress-driven elements, LRM/BM based modeling is proven available for linear SMA spring (Velázquez et al., 2006; An et al., 2012). However, for works that rely on large-shear-strain-driven SMA elements such as torsional wires, strips, rods, and tubes, full study on actuator multi-physical modeling cannot be found in the literature. Available researches focusing on modeling of thermomechanical behavior are given as follow: for reduced-order phenomenological model, exact solution based on BM and Lagoudas models can be found in works presented by Prahlad and Chopra (2007), Chung et al. (2006) and Mirzaeifar et al. (2010). A reduced-order mechanical model is given

$$\gamma =\frac{\theta r}{L}$$ (5)

$$\tau =\frac{Mr}{J}$$ (6)

$$J=\frac{\pi r^{4}}{2}\text{ for rods},\;J=\frac{\pi }{2}\left(r_{outer}^{4}-r_{inner}^{4}\right)\text{ for tubes}$$ (7)

with *γ*, *τ*, *θ*, *M*, *J*, *r*, *L* the shear strain, shear stress, rotation angle, subjected torque, polar second moment, radius and length of actuator, respectively. Experimental data of torsional SMA elements can be found in (Prahlad and Chopra, 2007; Chapman et al., 2011; Doaré et al., 2012; Rao et al., 2014), the results shown that reduced-order models combined with Eqs 5–7 sufficiently predict the behavior of thin-walled tubes and thin rod/wire with diameter up to 6 mm. A 3D FEM model offers more accurate results for thick wall tubes or thick rods. Work by Zhu et al. (2013) shown that results are given based on Lagoudas, Brinson, and Auricchio model in 3D FEM framework yield similar global torque-angle relation for a pure torsional motion for a tube of 20 mm in diameter and 1.5 mm in thickness (see in Figure 10A). Figure 10B shows simulation results of the 3D FEM model and experimental data of PE behavior of three SMA torsional wires with the same length and different diameters around 0.5 mm provided in work by Chapman et al. (2011). The results show that the 3D FEM model can accurately capture the influence of design parameters on global response with a high hysteresis. Besides the analytical approach, the stress-strain experimental data can also be used for the prediction of actuator performance (Firouzeh et al., 2013; Zhakypov et al., 2017).

To sum up, for multiphysics modeling of active bias actuators, currently, the most popular strategy is directly using the existing 1D models, like the classical LRM or BM coupled with thermal balance equation (see Table 2). So far, this approach has led to interesting results. However, it does not fully describe the response for more complex geometries such as bending sheets and torsional elements. The existing applications demonstrated active bias torsional elements’ capacity, but complete modeling of its nonlinear response is still on demand.

### 5.2 Actuators Characterization

The characterization of actuators is presented in two parts: 1) characterization of elementary SMAs, and 2) characterization of actuation systems. Focusing on elementary SMAs, two tests are frequently mentioned according to model parameters identification and test simplicity: the isobaric test and the isothermal tests. The former is to capture the hysteresis at constant tensile force or torque with cooling-heating cycles. The latter is to capture the PE behavior with constant high temperature using loading-unloading cycles. With these tests, the SMAs element’s material parameter and performance can be identified. Other tests, such as the blocked strain test that combine the “Austenite curve” using different constant strain,^11^ fatigue test for accumulation of permanent strain during cyclic loading and power consumption tests can be found in the literature but will not be discussed in this work. One need to remind that these tests are normally taking place using a wire form simple (Prahlad and Chopra, 2007; Chapman et al., 2011; Guo et al., 2013; Doroudchi et al., 2018), but parameters directly identified from a torsional test are shown to be acceptable for work with unconventional form element (Zhakypov et al., 2016). However, several technical problems need to be addressed due to measurement difficulties. In the work of Churchill et al. (2009), it is indicated that the results of SMA wires tests highly rely on specific factors such as temperature controlling, loading methods, and measurement techniques. For example, unlike conventional metals, where temperature variation can be tolerated without influencing the results’ accuracy, an SMA wire’s response can be significantly affected by a few degrees of change in sample temperature. Thus temperature sensing and controlling for a themo-electrical effect-based SMA are very difficult. Such problems will also be validated in the case of torsional elements. Consequently, experimental results of the millimeter-sized torsional elements are minimal than that of centimeter size. An introduction of the phenomena that can lead to testing problems and technical solutions for accurate qualifications is challenging. Focusing on BRM actuators, investigations based on two key performances can be found in the literature, such as the motion range and the output torque. The former is relying to the capability of local motion, which is the main objective in robotic and medical domains. The latter is often the mean objective of works in the aerospace and automobile domain. Based on the review, the bending cantilever offers a lower stroke than the other design. The “linear + pulley” design offers a maximum rotation angle of 180° but highly depends on the pulley’s radius and requires large operation space. However, the supplementary mechanism has been frequently mentioned in the literature and has been identified as a source of friction. The antagonistic torsional elements arrangement provides a larger stroke than others designs without any supplementary mechanism (Koh et al., 2014). It is important to mention that the actuators are attached to an origami-inspired paper-based open structure. Thus the output torque has not been characterized. Base on Table 2, characterizations are usually focusing on SMA elements performances and system motion range. The strategies and methods for system output torque measurement are still in demand.

### 5.3 Actuators Performance Prediction and Designing

The SMAs’ behavior importantly depends on its thermomechanical loading path due to its high hysteresis. In fact, since SMAs elements usually need an external bias force for re-initialization of shape, the understanding of the stress–strain-temperature dependence becomes extremely important for the choice of design parameters. Generally, the capability due to SME of an SMA according to a certain temperature is well described by its “M→A curve” so-call “Austenite curve” (see in Figure 3E), which can be identified and modeled using the method described in Section 5.2.

**FIGURE 11:**
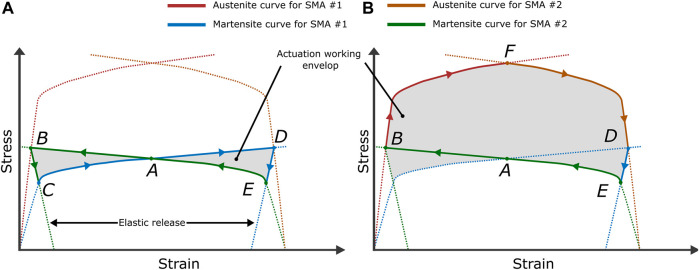
Example of active biased SMA performance tests: **(A)**: motion range tests, the (B–D) presents the maximum of motion range. **(B)**: maximum output stress tests, the F presents the maximum output stress of system at certain temperature. The Panel is inspired by Georges et al. (2012).

Figure 11 shown a stress-strain relation of a typical actuation system defined by two identical SMA active elements and pre-deformed at the same level. The blue and green curve presents the inelastic behavior according to pure martensite state, and the red curve shows the “Austenite curve” at a certain high temperature. The Figure 11A shown a typical bidirectional actuation configuration, the activated SMA only needs to overcome the reaction of its cooled antagonist component, then finish the activation at the equilibrium point (B), and follow by an elastic release during cooling with a definitive equilibrium as soon as the power is off. Then the strain between (B) and (D) presents the motion range of the actuator, and the (C) and (E) present the equilibrium point at the martensite state. The figure on the right shows the scenario of providing maximum output stress of SMA #1 to conquer the stiffness provided by the activated SMA #2. Using this tool, users can rapidly establish primary design parameters using experimental data (Firouzeh and Paik, 2015). Additionally, inverse model using identified parameters (for example in Figure 10B) can be generally used for determining the design parameters according to application requirements. For example, A non-rigid Kresling-pattern-based origami structure so-call “Kresling tower” can perform a shape morphing using a global actuation (Jianguo et al., 2016) at the tower’s center-line or local actuation at the “hinge-like” folding creases (Kim J. et al., 2015). The former usually requires higher torque to overcome the structural stiffness (Li et al., 2019), and the latter requires a larger rotation angle. Certain design constraints should be carefully taken into account to avoid the degradation of the actuation system. In fact, The SMAs can be damaged with excessive mechanical load or thermal load (Mohd Jani et al., 2014). Many researchers have concluded that thermal load is an important factor of the determination of Ni-Ti based SMAs’ fatigue performance. Overheating reduces the fatigue life of SMA actuators (Mohd Jani et al., 2014) significantly and damages the structural material. To ensure the applications are intended to perform securely over countless cycles (around $10^{6}$ cycles), the condition such as maximum function temperature, the maximum strain (Huang, 2002; Dynalloy, 2011) and the safe design load (Reynaerts and Van Brussel, 1998) should be carefully decided and consequently utilized as the feasible domain of optimization of design parameters.

### 5.4 Activation and Cooling Strategies

**FIGURE 12:**
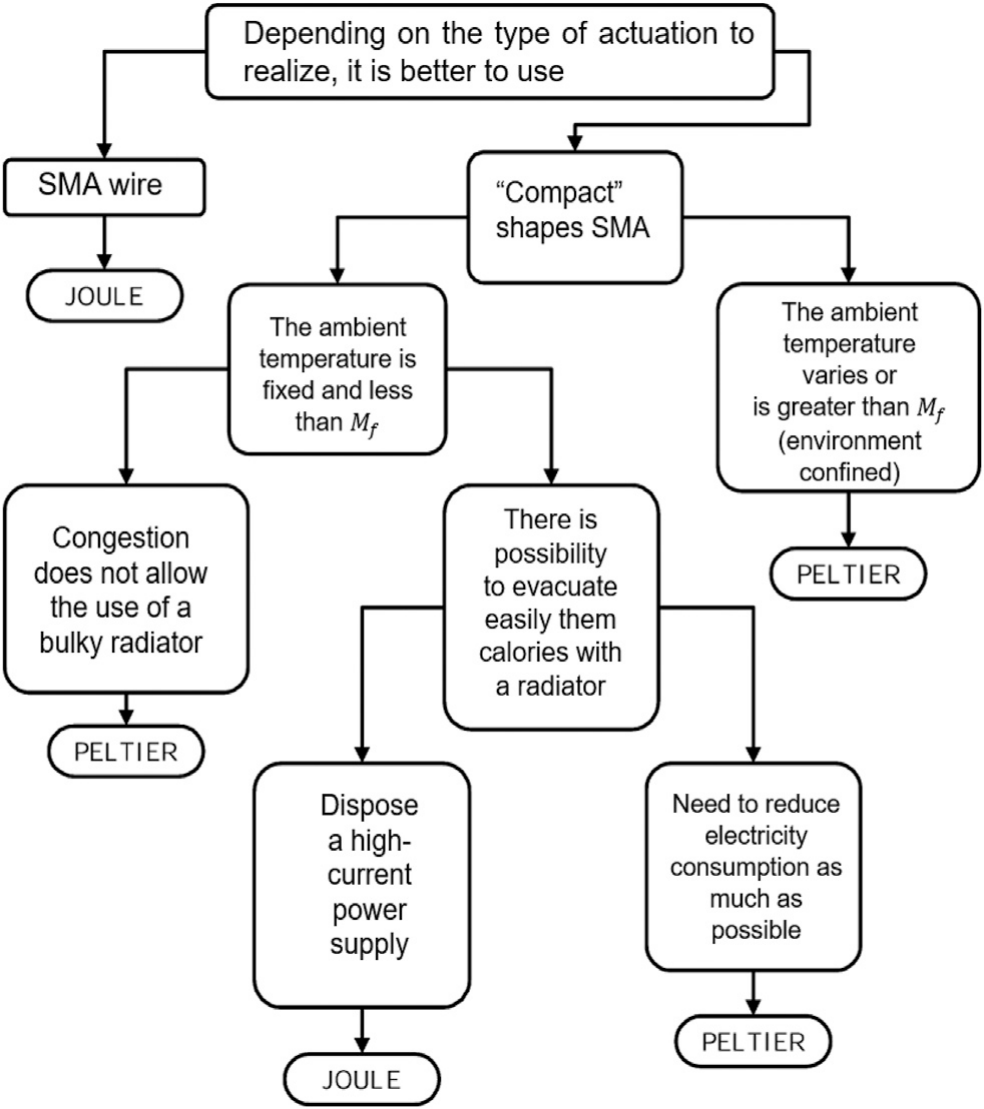
A guideline for determination of activation method of SMA elementary actuator between Joule effect and Peltier effect (Abadie, 2000).

The heating and cooling path is an essential requirement for SMAs’ mechanical response. The former relies on the SMA activation due to the “M→A” transition, and the latter relies on the “re-arm” process of actuators. In fact, low-speed response and low energy efficiency are two of the main SMA disadvantages (Paik et al., 2011; Mohd Jani et al., 2014; Doroudchi et al., 2018). Various thermal power supplying and removal methods can be found to this end. Concerning SMAs activation, strategies are given as:

1) Joule effect heating using the actuator body as the resistance in the electrical circuit. Consequently, a higher current offers higher activation speed. In fact, this method is proven as the best method for low-diameter normal-stress-driven SMA because of its simplicity and controllability. However, the high current can cause problems for associated electrical traces due to $I^{2}R$ losses, where *I* and *R* are the current and electrical resistance, respectively.
2) External heater-cooler using thermoelectric effects such as the Peltier effect, the Thomson effect, and the Seeback effect (Shahin et al., 1994; Abadie et al., 2002). The electrical current applied to the module can heat or cool the SMA element of the actuator. Studies based on this method are presented in (Abadie et al., 2002; Abadie et al., 2009). An SMA blade-form actuator realized by a thermoelectric system composed of two bismuth tellurides (${\text{Bi}}_{2}{\text{Te}}_{3}$) ingots is tested, resulting in an improvement of response dynamic during cooling. The Figure 12 shows a guideline to determinate the activation method.
3) External heater using thermal conduction often consists of resistance wire or thin layer. This method offers a solution to overcome the low electrical resistance due to actuators’ design and SMAs’ material properties. Works presented by Paik offer an analysis focusing on the designing of an external heater made of Ni-Cr alloy (as called “Inconel”) with different forms, such as a coil form wire heater (Paik et al., 2010), or a thin-film layer form with a 2D pattern heater (Paik et al., 2012; Zhakypov et al., 2016). The results showed that this Ni-Cr heater offers a 20% reduction in response time and around a 53% reduction in power consumption than a Joule-effect-based heater.

To sum up, the Joule effect-based heating is proven a common method for SMA activation. The electro-thermal effect-based heating and thermal conduction heating showed a better performance in terms of activation time or consumption efficiency regarding specific actuator geometries. On the other hand, the low cooling rate is indicated by many researchers as one mean drawback of SMA. As indicated by Jani et al. (2014), the SMAs have a relatively high heat capacity and density, resulting in lower heat transfer rate and operation bandwidth problem. Base on the literature, the natural air convention seems a common way for SMAs’ cooling. One needs to mention that the actuator response time is affected by their size and shape, where ones with lower diameters cool faster due to their higher surface-to-volume ratio. Beyond this method, improvements due to convective condition (force air or liquid convection) and to conductive material (heat skin) were carried out in the literature (see Table 2). One need to mention that the modular introduced by Abadie et al. (2002) offered improvement in response time both in heating and cooling. The actuator provided a ten times greater deflection than using the Joule effect and air convection with 1 Hz.

### 5.5 Sensing and Controlling Methods

The lack of control accuracy is one of the main disadvantages of SMA actuators. Since the electro-thermal method is usually implemented to activate actuators, the measurement of the actuator’s real strain is difficult. Based on the literature, a rotary mechanism embedded with an encoder DC rotary motor is a standard method to measure the actuation system’s motion range using SMA wire. For SMA in other forms such as bending sheets or torsional springs, the image processing is often implemented, and it is proven to be a suitable method for motion sensing (see Table 2). Beyond this two common methods, one needs to highlight two approaches on sensing of SMA for BRM:

1) SMA wires can be used as self-sensing actuators because its unique hysteresis: the variation of its electric resistance R and the strain of actuator *ϵ* yield a linear relation due to the changing of its geometry (Ruth et al., 2015; Prechtl et al., 2020);
2) A stretchable mesoscale bending sensor named “elastic curvature sensor” is presented in (Firouzeh et al., 2013), it is manufactured using carbon impregnated silicone rubber and it is capable of offering a repeatable measurement with a rotation angle up to 150°.

Focusing on controlling methods for BRM application, different methods can be found in the literature. For example, the PI/PID controller have been frequently implemented and have been proven capable for the accurate control of SMA-wire-based actuators [(Moallem and Tabrizi, 2008; Ruth et al., 2015; Guo et al., 2013, 2015; Georges et al., 2013; Doroudchi et al., 2018), for normal-strain-driven application (Sheng and Desai, 2015; Sheng et al., 2017), for torsional spring (Shin et al., 2016; Kim Y. et al., 2019), for bending wire]. The controlling performance are presented in the Section 4. However, as indicated by Gédouin et al. (2011), a difficulty is that the properties of the material vary during cyclic loading. Consequently, the stiffness of SMA varies during cyclic applications, thus induces complexities in the classic control model. To construct an easy-to-use (without the physical model) and robust controller, non-physical model-based controlling model so call “physical model-free control” was introduced by Kha and Ahn (2006), Gédouin et al. (2011), Ashrafiuon and Elahinia (2015). It is worth mentioning that Ashrafiuon and Elahinia (2015) performed a detailed investigation of a sliding model control (SMC) (Elahinia and Ashrafiuon, 2002). In this article, both non-model-based and model-based (SMC and LRM) robust control methods were implemented for the control of a three DOF (rotary joints) SMA actuated robot arm using 2 two passive biased SMA wire. The prototype was able to provide around 100° rotation with an error smaller than 0.5°. The model was applied in works of (De Sars et al., 2010; Ruth et al., 2015; Moghadam et al., 2019) and the results showed that the combination of constitutive model and SMC offers an improvement of actuator’s control accuracy of angle than the classic PID controller (Moghadam et al., 2019). One needs to remind that these models were applied to normal-motion-driven SMAs. Thus a study that couples the SMC and bending or torsional motion-driven SMAs seems interesting. Beyond the controller for BRM actuators, approaches on SMA-based control algorithm such as pulse width modulation (Gharaybeh and Burdea, 1994), fuzzy logic (Kha and Ahn, 2006), neural networks (Song et al., 2003), and variable structure control (Grant and Hayward, 1997) are found in the literature but will not be discussed in this work.

## 6 Conclusion and Perspectives

This review article summarizes the different types of SMA-based bidirectional rotational motion actuation, where the one-dimensional deformation of the SMA element can be transferred into rotational motion in opposite directions using various arrangements and temperature control methods. As identified in this review, SMA can play a significant role in realizing BRM in multiple-scales. Classifications are carried out based on various features: elementary SMA for uni-directional rotational motion; passive and active biased actuators for different types of rotational articulation of BRM. The article has presented various summaries of solutions for BRM actuation using thermal-activated SMA elements. The torsional SMA element showed an exciting potential for an application that needs a large rotation angle within limited operational space, such as origami-inspired robotics. Based on the review, discussion regarding various aspects is carried out.

On the other hand, to enhance this exciting approach, several challenges need to be addressed: Development and improvement of analytical tools for actuators prediction constitute the first challenge. Indeed, very efficient multi-physical reduced-order models have been developed for actuators based on simple geometries, but rigorous analyses are required so that they can be applied for reliably capturing the thermo-mechanical response of actuators with complex geometries. Moreover, in addition to the non-linearities associated to the material behavior, origami structures involve highly nonlinear behaviors due to the changes in geometry, that should be considered in the upcoming development of these design tools.

The second challenge is related to BRM actuators design. The stress-strain plane using the austenite curve of the active element and the hosting structure is frequently mentioned as a valuable and effective tool. Previous works show that this method is acceptable not only for a passive biased SMA actuator but also for antagonist configuration. However, the method is based on a fixed dimension SMA element and the studies usually focus on designing the bias element (for example, a passive biased spring with different stiffness). Regarding the technical demands for origami robots, working conditions such as actuators’ dimension, arrangement method, and temperature conditions need to be addressed: design tools based on inverse problem that takes all those factors into account would constitute an added-value for the community.

The characterization of torsional actuators constitutes another challenge that needs to be addressed. Although a few experimental methods are available (as detailed in Section 5.2) for torsional SMA elements, the results according to millimeter-sized actuators which are required for a wide variety of origami-based robots are still very limited compared to actuators in centimetre-size. The results of SMA tests highly rely on specific factors such as temperature controlling, loading methods, and measurement techniques. Thus, an investigation of the phenomena that can lead to testing problems and technical solutions for accurate qualifications for millimeter-size actuators is challenging. On the other hand, the characterization results of existing BRM actuators usually focus on SMA elements performances and system motion range. Those results are not sufficient for self-folding structures that need both output torque and angle stroke to achieve shape-changing. Thus, the strategies and methods for system output torque measurement are still in demand.

The next challenge is related on actuators’ activation and cooling. The Joule effect-based heating has become a standard method for SMA activation. The electro-thermal effect-based heating and thermal conduction heating have shown better performance in terms of activation time or energy efficiency, regarding specific actuator geometries. On the other hand, many researchers have identified the low cooling rate as the main drawback of SMA-based actuators. Specific improvement can be found in the literature, but a systematical investigation of cooling methods for SMA-based torsional actuators is still in demand.

Challenges related to the control of BRM actuators are still pressing. The combination of SMA constitutive model and PID control was proven suitable and applied as a common controlling method for BRM actuators. Moreover, compared to this approach, the sliding-model-controller (SMC) with or without SMAs’ physical model showed improvements in response time and control accuracy, respecting the linear SMA element. Finally, studies that couple the SMC and bending or torsional motion-driven SMAs are required. Perspective can be a complementary study of BRM actuator using active biased torsional SMA element and integrating such actuator for origami structure’s shape-changing and shape blocking.

## Footnotes

1 Nitinol was first discovered by Buehler et al. (1963), it is known as a conventional SMA.

2 Up to 10^6^ $10^{6}$ action cycles under a strain of 1%.

3 Twinned martensite, detwinned martensite, and austenite.

4
$\sigma_{s}^{\text{cr}}$ is far lower than the permanent plastic yield stress of martensite.

5
*ξ*: the martensite volume factor, 0% presents pure austenitic state, 100% presents pure martensitic state.

6
$\xi_{T},\xi_{S}$ : the martensite volume factor induced by temperature and the martensite volume factor induced by applied stress.

7 Compared to a conventional torsional strain-based application, such as SMA linear springs (An et al., 2012).

8 These two values are according to the begin and the finish state of “M→A” transition.

9 To maintain the desired shape without additional force after actuation.

10 A that can be melt at relatively low temperature while avoiding the degradation of the device, the material is heated by a commercial Ni-chrome wires

11 For example, using similar electrical input condition with different blocking position, using measured torque at the same time to construct the “Austenite curve” (Zhakypov et al., 2016).
